# Exploring Binding Properties of Agonists Interacting with a δ-Opioid Receptor

**DOI:** 10.1371/journal.pone.0052633

**Published:** 2012-12-26

**Authors:** Francesca Collu, Matteo Ceccarelli, Paolo Ruggerone

**Affiliations:** 1 CNR-IOM SLACS and Dipartimento di Fisica, Università degli Studi di Cagliari, Monserrato, Italy; 2 Dipartimento di Fisica, Università degli Studi di Cagliari, Monserrato, Italy; King's College London, United Kingdom

## Abstract

Ligand-receptor interactions are at the basis of the mediation of our physiological responses to a large variety of ligands, such as hormones, neurotransmitters and environmental stimulants, and their tuning represents the goal of a large variety of therapies. Several molecular details of these interactions are still largely unknown. In an effort to shed some light on this important issue, we performed a computational study on the interaction of two related compounds differing by a single methyl group (clozapine and desmethylclozapine) with a 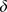-opioid receptor. According to experiments, desmethylclozapine is more active than clozapine, providing a system well suited for a comparative study. We investigated stable configurations of the two drugs inside the receptor by simulating their escape routes by molecular dynamics simulations. Our results point out that the action of the compounds might be related to the spatial and temporal distribution of the affinity sites they visit during their permanency. Moreover, no particularly pronounced structural perturbations of the receptor were detected during the simulations, reinforcing the idea of a strong dynamical character of the interaction process, with an important role played by the solvent in addition.

## Introduction

G-protein coupled receptors (GPCR) communicate signals across cell membranes in response to an astonishing variety of extracellular stimuli - light, proteins, peptides, small molecules, hormones, and ions, and have been found to activate other, G protein-independent, signaling pathways. The particular response of the system is determined by the nature of the ligand-receptor interactions. However, questions on the nature of ligand-induced changes determining and on the extent of structural features responsible for receptor activation/inactivation remain still obscure and need to be clarified. The importance of including as much as possible microscopic details and structural features has guided improvements and adjustments of models and techniques used to describe and to study ligand-receptor interactions [1], [2], from the simple lock-and-key picture to the induced fit theory. Systems that represent a valuable and challenging test for such techniques are opioid receptors, a family of GPCRs, which are the target of a large variety of drugs. They are integral membrane proteins of the central nervous system implicated in mediating the analgesic effects of opium derived alkaloids, endogenous ligands such as the enkephalins, and their precursors [3]–[6]. Opioid-receptor binding and activation processes for the purposes of drug design are still not fully understood. This knowledge might be crucial for a rational drug design aiming at the identification of more potent and efficient compounds with fewer or no adverse side effects, such as respiratory depression [7], development of tolerance and dependence [8], nausea [9] and constipation [10].

**Figure 1:**
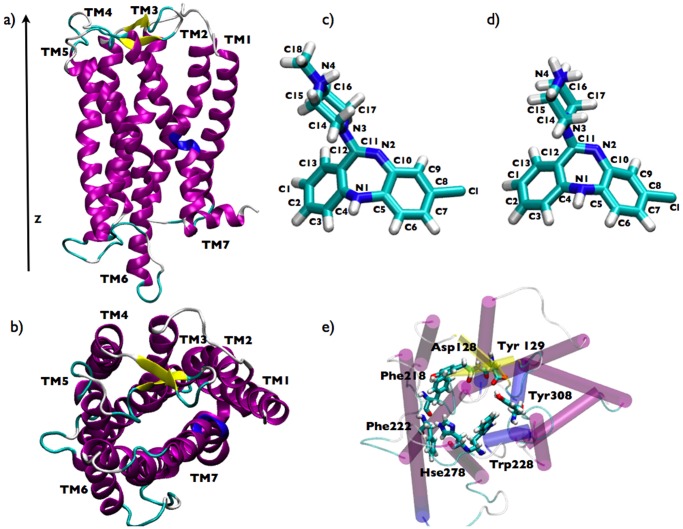
View of the systems under study. (a) side and (b) top view of the homology-modelled 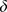-opioid receptor; licorice picture of (c) clozapine and (d) desmethylclozapine; (e) top view of the putative active site of the 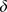-opioid receptor and the associated residues.

The lack of a crystal structure has hampered our understanding, in general, of the structure-dynamics-function relationship and, in particular, of the action of specific natural ligands and artificial compounds on opioid receptors and several other GPCRs. To overcome this drawback, structural models of the ligand-bound system have been developed by exploiting different computational techniques mainly applied on the crystal structures of rhodopsin [11] and more recently of 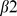-adrenergic receptor [12] as template (see Pogozheva *et al.* [13] and Fanelli and De Benedetti [14], [15] for a review thereon). In parallel, computer simulations have been extensively used to investigate GPCRs for which a crystal structure exists [16]–[25]. Among GPCRs, the 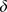-opioid receptor (see Figure 1, panels a and b) is an especially attractive target in the development of new drugs for the control of pain. For example, compared to compounds targeting other opioid or opioid-like receptors, 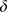-opioid-selective drugs have certain advantages, including: greater relief of neuropathic pain, reduced respiratory depression and constipation, and reduced potential for the development of physical dependence [26]. In the absence of a crystal structure of 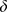-opioid receptors (only very recently a crystal structure of a 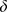-opioid receptor interacting with an antagonist has been published [27] at the same time of the release of the structures of 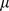-opioid [28] and 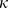-opioid [29] receptors) information at microscopic level on the functioning of opioid receptors has been gained through computational studies [30]–[34]. In their works Bernard et al. [30], [31] used molecular dynamics (MD) simulations as a mean to sample the conformational space of ligands in order to include all accessible conformers at room temperature in the development of a pharmacophore. Provasi and coworkers [33], [34] focused their attention on the possible assembly of 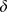-opioid receptors in form of homodimer. Their computer results indicated such homodimers have a short lifetime in the membrane. Concerning the interaction of a ligand with a 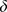-opioid receptor, Provasi et al. explored the binding of the nonselective antagonist naloxone in a human 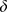-opioid receptor via a thorough computational study based on diverse metadynamics approaches [32]. They suggested a preferential entry pathway of their ligand starting at a molecular recognition site on the surface of the 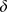-opioid receptor and ending in the receptor alkaloid binding pocket. These successful applications of simulation techniques to the problem of ligand-receptor interactions have paved the way to more extended computational investigations, and the possibility to compare simulation results with the recently available crystal structure of 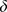-opioid receptor [27] represents an additional step toward the understanding of opioid receptor functioning.

In the present work we complemented and extended the previous studies on the ligand-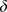-opioid interactions using molecular dynamics (MD) simulation.

To shed some light on the aspects related to the ligand-receptor interaction we investigated the behaviour of two agonists: clozapine (CLZ) and N-desmethylclozapine (DSM). CLZ is the prototype of a group of atypical antipsychotic drugs exhibiting clinical efficacy similar to that of the classical antipsychotics but not producing, or inducing to a lesser extent, most of their motor side effects, such as catalepsy [35]. DSM is one of the major metabolites of clozapine [36] and differs from it for the lacking of a methyl group. DSM has become a focus of interest in its own right with regard to its biological activity and its potential therapeutic use, because it shares the CLZ ability to bind to a large array of neurotransmitter receptor systems. Additionally, DSM activates 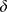-opioid receptors endogenously expressed in brain while in the brain membrane preparations CLZ displays negligible agonist activity [35], [37]. Determination of intrinsic efficacies by taking into consideration both the maximal [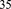
S]GTP
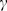
S binding stimulation and the extent of receptor occupancy at which half-maximal effect occurred indicated that DSM has an efficacy value equal to 82% of that of the full 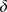-opioid-receptor agonist DPDPE, whereas CLZ displays much lower levels of agonist efficacy [37], [38]. Since the two compounds differ in a methyl group, they are particularly well suited for a comparative study, homing in on understanding the microscopic details of their interactions with the target. Acquiring this information may represent a good starting point to rationalize the drug design. Computer simulations have the required temporal and spatial resolutions to enlighten the microscopic features described above, and even the penalizing time gap between simulation and real time scales can be bridged in several cases due to the development of algorithms able to describe rare events. One of these methods, the metadynamics [39], [40], was used in the present work to mimic the exit of CLZ and DSM out of the 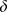-opioid receptor. According to our results the better activity of DSM might be attributed to spatially and temporally extended interactions of the compound with the receptor, instead of a pronounced interactions with a limited number of residues. In other words, the binding mechanism of the ligand inside the receptor acquires, in our view, a more dynamical (including role of the solvent) than purely structural character, supporting the idea of Wacker *et al.* based on the analysis of the X-ray structure of the 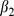 adrenergic receptor interacting with different ligands [41]. Additionally, the diverse regions inside the receptor explored by CLZ and DSM seem to map well the structural determinants of *message* (efficacy) and *address* (selectivity) [42], [43] identified by Granier et al. [27].

In our picture a stabilizing effect is played by *long-time resident* waters (for times of the order of 1 ns) present in the locations visited by DSM but absent in those explored by CLZ. The pivotal role of solvent in the activity of GPCRs has been already pointed out by several works [16], [17], [19]–[22] and the picture coming out from our work is consistent with the evidences reported in the literature. It is worth pointing out that our results for the 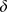-opioid receptor combined with those extracted from experimental and theoretical studies on other GPCRs provide a more general picture of affinity of the receptors toward a ligand based on a deep and still not completely enlightened interplay among structural and dynamical features and interaction with the solvent.

## Results

### Dissection of the free energy surfaces

**Figure 2:**
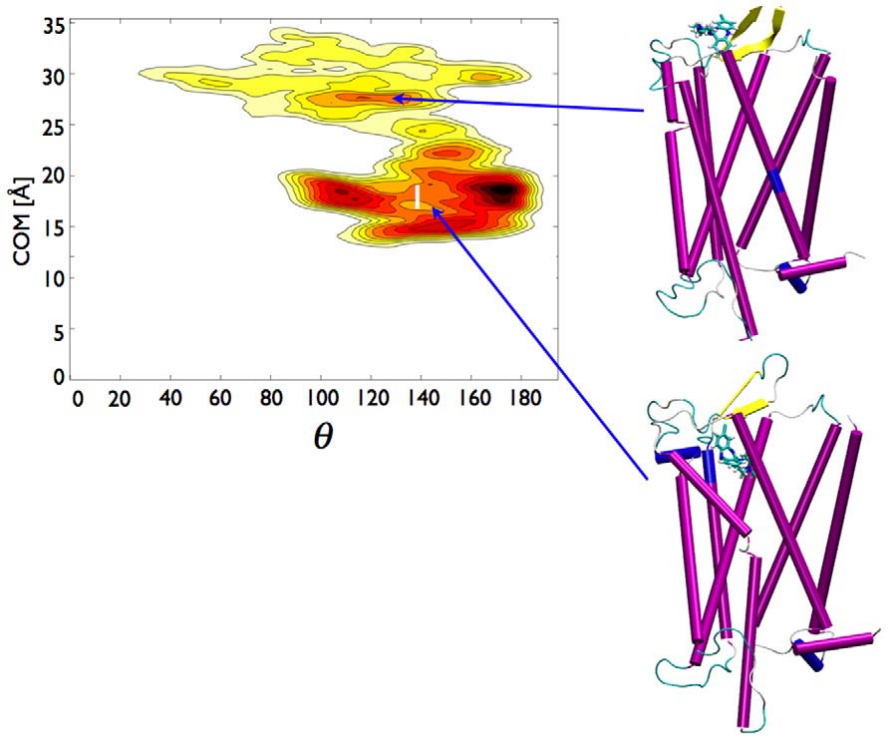
Free energy surfaces obtained from metadynamics simulations for CLZ as a function of d 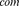 and 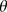. Each line corresponds to 1 kcal/mol. A snapshot of the systems extracted from standard MD simulations in the region labelled by 1 is also shown. See text for discussion.

**Figure 3:**
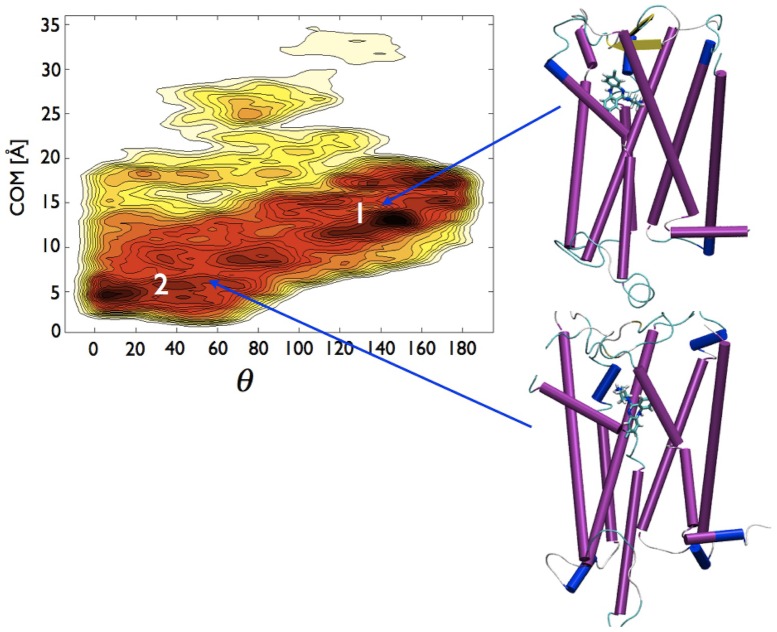
Free energy surfaces obtained from metadynamics simulations for desmethylclozapine as a function of d 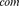 and 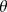. See Figure 2 for further details.

**Figure 4:**
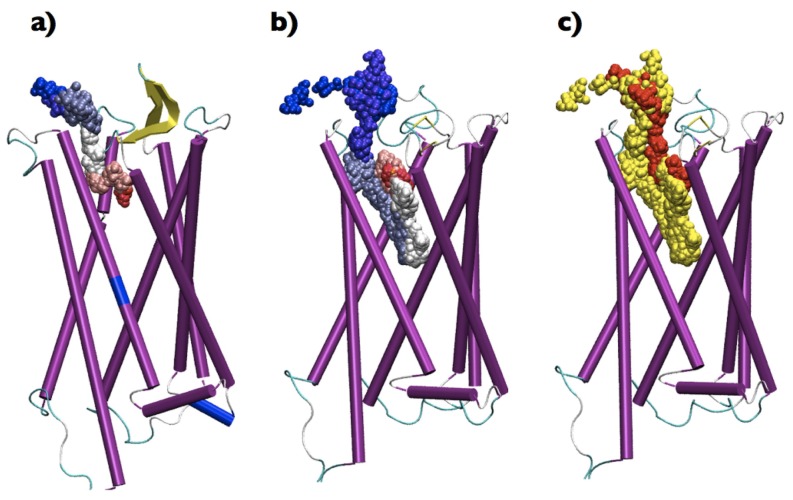
Bead representation of the time evolution of (a) CLZ and (b) DSM centers of mass during metadynamics. The initial beds are colored in red, the final in blue. (c) Superposition of the two series of beds, in yellow DSM, in red CLZ.

The free energy surfaces (FESs) associated with the escape process of CLZ and DSM are represented in Figures 2 and 3, respectively, as a function of the two coordinates we chose to accelerate during the metadynamics runs, namely the distance between the centers of mass of the compound (either CLZ or DSM) and of the receptor, d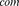, and the orientation of the axis of the compounds, identified by the N1 and N4 atoms, with respect to the receptor axis, 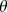 (see *Materials and Methods*). Initially, CLZ and DSM were located inside the receptor in the positions determined as described in *Materials and Methods*. In these positions both compounds interacted with Asp128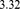, an important residue involved in the recognition of biogenic amines by GPCR [44], [45]. Note that residue numbering here and throughout the text follows the Ballesteros-Weinstein notation [46]. According to this notation, each residue is indicated by a two-number identifier N1.N2 where N1 is the number of the transmembrane helix, and N2 is the residue number on that helix relative to its most conserved position. The two FESs remarkably differed in shape and extension. A part a common region explored by the two compounds (cfr. Figure 4) DSM sampled a more extended portion of the configuration space defined by the two variables than CLZ did: 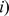 DSM reached very deep sites of the receptor corresponding to d
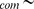
2–3 Å, while the lowest value of d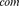 for CLZ was 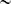
15 Å; 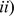 the range of orientations sampled by DSM was wider than the one explored by CLZ. For d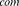 comprised between 5 Å and 18 Å DSM assumed practically all the possible values of 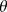. Instead, the range of CLZ orientations was very narrow spanning the interval (90°,180°) in the region where the affinity sites were located. Note that the different shapes of the FESs for d
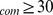
Å were due to to the fact that the two compounds reached the value of d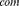, corresponding to the loop region and considered as the stopping limit of the metadynamics runs, at different times. Thus, the FESs for d
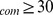
Å reflected the different time spent in this region by the two compounds.

The two FESs, especially the one associated with DSM, were characterized by several minima. However, standard MD simulations performed on the two systems starting from the deepest minima of the FESs, suggested to speak more appropriately of affinity basins for the two compounds. The 70 ns-long MD trajectories touched entire regions encompassing more than one minimum instead of being confined in a single minimum. For CLZ a unique affinity Basin, labeled by 1 in Figure 2, contained the entire region with d
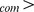
20 Å. DSM moved between two distinct affinity Basins, 1 and 2 in Figure 3, the first centered at a slightly lower d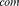 than the single Basin of CLZ, the second definitely in a very deep region of the receptor. We cannot rule out that CLZ might explore a second affinity Basin as DSM, but our results indicated that the access to this second Basin requires an activation barrier larger than the one necessary to move toward the exit of the receptor. According to the snapshots inserted in Figures 2 and 3 the orientations of CLZ and DSM in their respective Basins 1 were similar with their diazepine group directed toward the receptor exit. In Basin 2 the piperizine group of DSM pointed upward.

### Analysis of compound-receptor interactions

**Figure 5:**
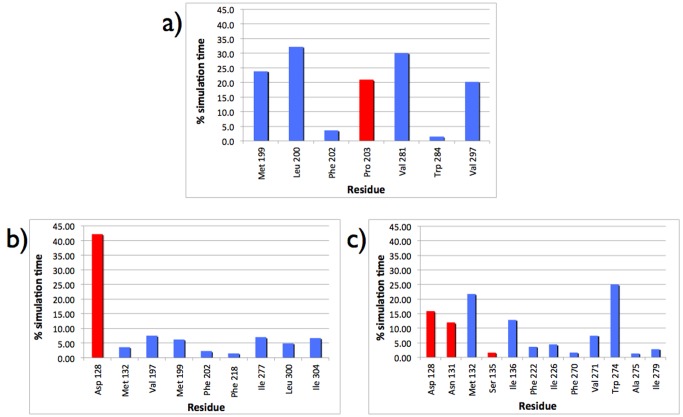
Statistical distribution (% of the total simulation time) of the direct interaction between residues and ligands in all Basins of the FESs. (a) CLZ in Basin 1, (b) DSM in Basin 1, and (c) DSM in Basin 2. Only the interactions observed for more than 1.5% of the simulation time are reported. Blue and red columns highlight hydrophobic contacts and H-bonds, respectively. The data are extracted from standard MD simulations lasting 70 ns in each Basin.

The analysis of the residues forming transient contacts (hydrophobic contacts and H bonds) with the compounds was carried out considering the trajectories extracted from the standard MD simulations in each affinity basin. Metadynamics was used to explore the free energy in the reduced space of the coordinates identified as relevant for the process under investigation. The biasing potential introduced in the system during the metadynamics runs might influence features of the system, including interaction pattern and solvent-related properties. Thus, the study of the latter properties required that the outcomes of metadynamics had to be complemented by standard unbiased MD simulations starting from the configurations assumed by the ligands in the relevant minima of the FESs. The statistical distributions of the residues having an interaction with the compounds detected at least for more than 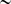
1 ns (corresponding to 1.5% of the total length of the simulation, i.e., 70 ns) are shown in Figure 5.

Statistically relevant H-bonds with the receptor were formed mostly by DSM (Figure 5 b and c) while CLZ was able to establish a H-bond only with residue Pro203 (Figure 5 a). Besides this H-bond CLZ made hydrophobic contacts with Leu200, Val281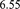, Met199, belonging to the External Loop 2 (EL2) and with Val281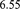 and Val297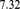. Asp128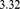, the important residue responsible of the recognition of biogenic amines by GPCR [44], [45], was H-bonded with CLZ but for a very short time, well below 1 ns. In Basin 1, as shown in Figure 5 b, the interaction pattern of DSM was dominated by the H-bond with Asp128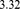, and the hydrophobic contacts were statistically less frequent. In Basin 2 (Figure 5 c) we observed a more homogenous distribution in terms of residues and nature of the interaction: hydrophobic contacts as well as H-bonds contributed nearly equally to stabilize DSM. The statistically more relevant contacts involved Trp274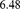,Met132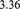, Ile136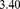 (hydrophobic contacts), and Asp128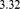 and Asn131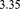, both H-bonds. Figure 5.

A further indication of the different affinity of the two compounds to the receptor came from the evaluation of their free energies of binding (see *Materials and Methods*). The configurational entropies were calculated within the framework of the Quasi-Harmonic (QH) approximation [47]–[49]. QH approach is an approximate method and its limitations have been deeply reviewed [49]–[53]. Several improvements have been proposed and successfully used to capture also conformational transitions [51], [52]. In our case, the ligands were essentially confined in single energy wells (the Basins) and this represent a situation for which QH inaccuracies are reduced [51]–[53]. In the affinity Basins 1 and 2, the free energies of binding of DSM were 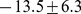 and 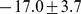 kcal/mol, respectively, whereas CLZ had in Basin 1 a free energy of binding of 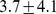 kcal/mol. According to our calculation the main contribution to this difference was of enthalpic origin (−25.0 kcal/mol and −29.6 kcal/mol for DSM in Basin 1 and 2, respectively, vs. −8.7 kcal/mol for CLZ), being the entropic terms essentially the same (11.5 kcal/mol and 12.6 kcal/mol for DSM respectively in Basin 1 and 2, and 12.4 kcal/mol for CLZ). The entropic contributions are the upper bounds of entropy, with the proper value of the entropy comprised between 0 and the QH value. Thus, although aware of the approximated character of the present evaluation and of the limited convergence of the entropic contribution, we are convinced that the difference between the free energies of binding calculated here supported the different interactions of the two ligands with the receptor.

### Analysis of solvent

An interesting aspect is related to the behaviour of the water molecules in all the minima identified in the FESs of the two compounds.

**Figure 6:**
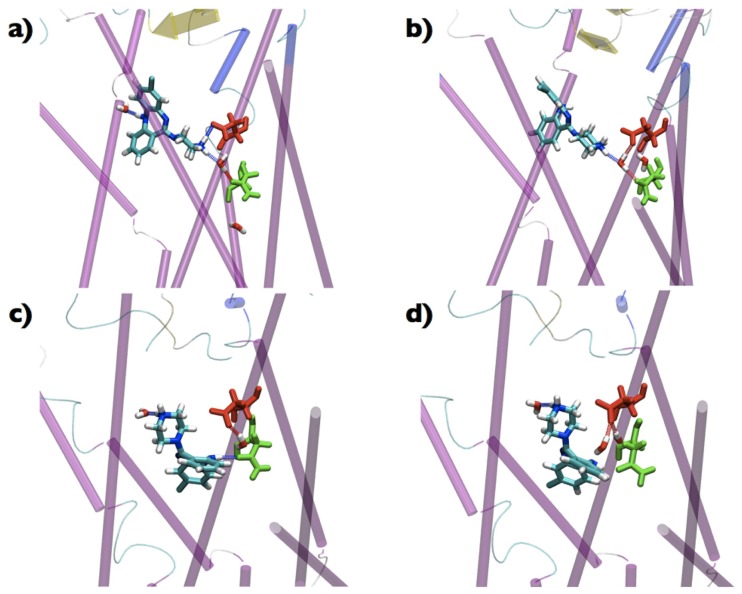
Interactions with solvent molecules. (a), (b), (c), and (d) snapshots extracted from a 70 ns-long standard MD simulation of DSM in Basin 1 and representing the long-time-resident waters interacting with DSM. In licorice we represent the two residues closer to the position of the waters: in red Asp128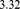 and in green Asn131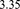. H bonds are shown as spring.

As for the determination of the interaction patterns, we used standard MD simulations to extract reliable and unbiased data for the study of solvent-related properties. The results of our analysis are collected in Table 1. As described in the Section *Materials and Methods*, the labels *fast*, *medium*, and *slow* refer to waters close to the ligand that have residence times of the order of 10, 100 and 1000 ps, respectively. These regimes were chosen following the literature and previous works of us [54]–[57]. DSM was able to form interaction with long-resident water molecules in both Basins, CLZ not. CLZ interacted only with fast and medium waters. In Figure 6 we report some snapshots extracted form the 70 ns-long standard MD simulations of DSM in Basin 1 and reproducing the behaviour of the two slow waters interacting with the compound. These water molecules formed H-bonds also with Asp128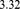 and Asn131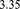.

**Table 1:** In depth analysis of ligand's solvation along each basins from 70 ns-long standard MD simulations.

	DSM				CLZ			
	Average number	Fast (%)	Medium (%)	Slow (%)	Average number	Fast (%)	Medium (%)	Slow (%)
Basin 1	10	38	42	20	14	73	27	0
Basin 2	9	78	20	2				
Water box	27	100	0	0	29	100	0	0

The average numbers of water surrounding DSM and CLZ are collected in the second and sixth columns, respectively, the values of fast, medium, and slow waters are expressed in percentage of the average numbers. To compare we reported the corresponding values for the two compounds inserted in a bulk water box extracted from a 15 ns-long standard MD simulation (third row).

### Ligand-induced perturbations

To gain deeper structural and dynamical insights into the interaction mechanism and, more importantly, to acquire a more reliable picture of the different effects induced by the compounds on the features of the receptor, we performed a thorough investigation of the stability of the different helices forming the receptor using TRAJELIX [58]. The post-simulation analysis concerned the trajectories extracted from standard MD simulations of 70 ns with the compounds located in all the relevant minima of Figures 2 and 3. We focused our attention on the helices that in the literature are reported as those mainly involved in the activation of the receptor, i.e., helices 3, 5, 6, and 7 [59]–[61]. No remarkable differences in the secondary structure of these helices were observed by inserting CLZ or DSM in the receptor (data not shown), pointing toward more dynamical than significant structural effects associated with the activity of the two compounds.

TRAJELIX made possible to monitor the time evolution of the following parameters of a helix: Tilt, Rotation, and Displacement. We evaluated these parameters for the apo receptor, and the receptor in complex with DSM and CLZ. For each case the parameters are defined with respect to a corresponding reference, which is in our case the equilibrated configuration of the system in the apo form and in complex with the compounds. In particular, Tilt is defined as the angle between the helix at a certain time and the same helix in the reference configuration, Rotation as the angle of revolution of the helix around its axis with respect to the reference, and Displacement as the displacement of the center of mass of the helix with respect to the reference. Of these local parameters we calculated from the trajectories the average values. To possibly relate these values to the activity we collected in Table 2 the change of the average values of Tilt, Rotation, and Displacement in the presence of DSM and CLZ with respect to the apo receptor.

**Table 2:** Variation of the average values of local structural parameters of helices 3, 5, 6, and 7 in the Basins of Figures 2 and 3 with respect to the analogous parameters of the empty receptor.

		DSM			CLZ		
		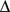 Tilt (°)	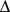 Rot (°)	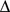 Disp (Å)	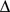 Tilt (°)	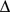 Rot (°)	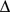 Disp(Å)
**Helix 3**	Basin 1	0.1 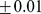	−2.1 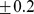	0.2 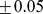	0.8 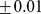	−1.6 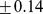	0.3 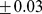
	Basin 2	0.5 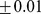	0.1 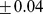	0.3 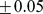			
**Helix 5**	Basin 1	0.6 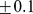	1.3 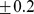	−0.3 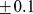	−0.7 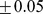	−0.8 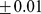	−0.6 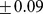
	Basin 2	2.7 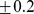	3.4 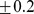	−0.2 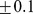			
**Helix 6**	Basin 1	0.7 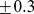	15.5 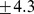	−0.5 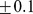	0.3 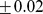	−0.4 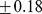	−0.7 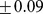
	Basin 2	2.0 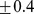	3.1 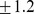	−0.7 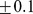			
**Helix 7**	Basin 1	3.1 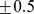	20.3 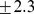	−0.5 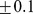	3.0 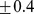	10.6 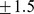	−1.1 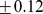
	Basin 2	5.9 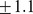	18.6 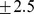	−0.1 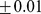			

The parameters are Tilt (Tilt), Rotation (Rot), and Displacement (Disp) and are extracted from the analysis of equilibrated 70 ns-long standard MD trajectories. Concerning rotation angle, negative values correspond to clockwise rotations, positive to anticlockwise. Standard deviations are also reported.

In Basin 1 the changes in the local parameters of helix 3 induced by DSM and CLZ with respect to the free receptor were nearly identical and generally small. Equally small were the changes of the same parameters when DSM was in Basin 2. For helices 5 and 6 the larger perturbations were induced by DSM, especially when the compound sat in Basin 2. Helix 7 was perturbed remarkably also by CLZ (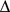 Tilt 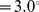 and 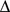 Rot 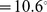) but DSM induced greater changes.

## Discussion

A first remarkable difference between CLZ and DSM is the shape of the respective FESs reported in Figures 2 and 3, respectively. According to the trajectories DSM samples two affinity Basins, CLZ only one. The methyl group seems to be a first discriminant of the possible regions explored by the two compounds and of the factors determining qualitatively and quantitatively the paths of the drugs inside the receptor. For CLZ, with its methyl group, the FES associated with the escape covers a reduced part of the (d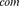,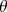) space and the values of 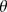 assumed by the ligand at the border between the protein interior and the loop region (d
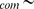
25 Å) are comprised between 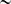
130° and 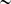
150°. The same access for DSM is larger in 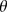 (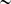
30°
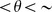
90°). The restriction of the orientations is due to the larger steric hindrance of CLZ compared to DSM: the methyl group limits the possible arrangements of CLZ within the receptor once the affinity Basin has been left. Thus, only a specific orientation of the compound permits the exit. Lacking the methyl group, DSM has a larger space to arrange itself within the protein. This explains the larger range of orientations explored by DSM. The presence of diverse regions of the receptor visited by the two compounds is in line with the outcomes of the very recent Granier et al.'s work [27]. They identified in their crystal structure of a 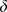-opioid receptor in complex with an antagonist distinct structural determinants of efficacy and selectivity of ligand binding. Within the framework of the *message-address* model of opioid receptor pharmacology the binding pocket of the receptor can be divided into two regions: the lower, highly conserved, is involved in recognizing the *message* of the ligand (efficacy), the upper, poorly conserved among opioid subtypes, is responsible of discriminating the *address* (selectivity). A correspondence between Basin 1 and the upper region of the binding pocket and between Basin 2 and the lower part might account for the enhanced activity of DSM compared to CLZ [37], [38]. Interestingly, although DSM and CLZ sample very close spatial regions associated with Basin 1 (see Figure 4), the list of above-threshold interacting residues extracted from Figure 5 does not overlap, i.e., there are no residues that interact with both compounds in Basin 1. Thus, the absence of the methyl group enables DSM to approach residues of the common region lining a side different from CLZ. This structural diversity affects also the interaction pattern. In Basin 1 DSM is not able to establish a wide network of interactions and the only remarkable linkage is the H-bond with Asp128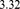, while Basin 2 is characterized by a large number of residues which the ligand is interacting with. A similar large distribution of stabilizing contacts is observed in Basin 1 for CLZ. The identification of two affinity Basins for DSM and a single one for CLZ is not at odd with the available crystal data on other GPCRs [12], [41], which identified a single binding site for several classes of compounds. First, according to our calculations CLZ and DSM had a common affinity basin (Basin 1); second, we did not rule out the possibility for CLZ to enter Basin 2 but our calculations pointed out that it was more likely for CLZ to move from Basin 1 out of the receptor than to explore Basin 2. A remark on the choice of the coordinates accelerated during the metadynamics runs is necessary. The choice of these coordinates is a very delicate issue for no proper criterion is available to guide it [62]. Our choice of (d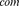,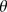) was driven by the geometry of the problem, although other coordinates can be selected. However, studies on confining systems such as DNA minor groove [63], bacterial pores [64], and also 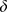-opioid receptor [32], used different sets of coordinates (including coordinates similar to those adopted in the present work) and demonstrated that the qualitative and quantitative features of a process involving narrow paths are not remarkably affected by the considered coordinates.

An indication of the microscopic reasons at the basis of the different activities of the compounds comes from the interaction pattern of the two compounds with single residues belonging to the putative active sites, i.e., Asp128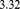, Tyr129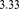, Phe218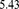, Phe222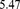, Trp274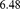, His278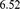), Tyr308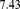 [65]. In the set of the residues statistically interacting more frequently with CLZ displayed, no residues of the putative active site appears whilst DSM is interacting with Asp 128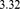 in both Basin 1 and Basin 2, with Phe218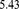 in Basin 1, with Phe222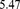, and Trp274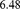 in Basin 2. Interestingly, durable interactions are established by CLZ with residues Met199, Leu200, Phe203, which belong to the External Loop 2 (EL2). EL2 was suggested as contributing source of stabilizing interactions with the nonselective antagonist naloxone in a metastable state [32]. Among the residues having a reasonable interaction with the compounds and belonging to the helices of the receptors we have the following situation: DSM is interacting with helices 3, 5, 6, and 7, while CLZ restricts its interaction to helices 6 and 7. The importance of specific helices in the activation of the signal has been pointed out in several works, and accordance has been reached in identifying helices 3, 5, 6, 7 as those taking the burden of activating the system [59]–[61]. Thus, a further support for the enhanced activity of DSM with respect to CLZ might be traced back to those preferred interactions established by DSM with the key helices of the receptor. However, it should be pointed out that there are no interactions of DSM with such putative relevant residues and helices with a pronounced statistical relevance, at least on the time scale sampled by our MD simulations. Apparently, DSM is not able to establish a particular relevant link with one of these key elements of the receptor, albeit it interacts with a larger number of them in comparison to CLZ. A dynamical picture of the ligand-receptor interaction comes out from our simulations. A confirm of a more dynamical than purely structural interaction pattern is apparent from the statistical distributions of the relevant interactions reported in Figure 5. As shown in Figure 5, CLZ has few remarkably long-lifetime interactions with some residues in Basin 1. DSM, on the contrary, a part the H-bonds formed with Asp128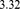, has a wide network of interacting partners with shorter life times as extracted form the MD simulations. Note that both compounds have a very weak or negligible interaction with residue Ile304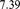. The relatively weak involvement of this residue in the binding affinity is supported by the observation that the I304T mutation did not reduce the binding affinity of a set of delta-selective ligands [66]. Differently, the same study pointed out that alanine mutations of Trp 284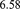 and Val 297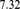 significantly weaken the binding of opioid alkaloids. According to Figure 5 CLZ has a remarkable interaction with Val297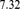 and a weaker with Trp284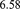 whilst DSM does not establish any noticeable interactions with the two residues.

Concerning the role of water molecules, Table 1 shows that CLZ interacts only with fast and medium waters, while DSM has a slow exchange with water molecules in both Basins, being Basin 1 the location with the maximum average number of water. This finding points out for DSM a stabilizing and mediating effect of water, which is absent for CLZ. These water molecules interact, according to our analysis, in addition with Asp128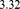 and Asn131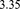, as it is possible to see in Figure 6, where snapshots extracted from the MD simulations are collected. Both residues belong to helix 3 and Asp128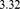 also to the putative active site of the receptor. Interestingly, the mediating effect of water is evident in Figure 6 a and b, where a water is establishing a linkage between DSM and either with Asp128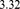 or Asn131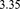. On the other side, in Figure 6 c and d one of the two waters is not interacting with residues of the receptor but only with the ligand. In any case, the interaction of DSM with the solvent molecules is either bridging the ligand directly to the receptor (residues Asp128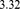 and Asn131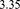) or extending the effective volume of the ligand. Such a complementing effect is not observed in our simulations when CLZ is inserted in the receptor. The presence of the long-time-resident waters is responsible for the more enhanced effects caused by the interaction of DSM with the receptor if compared with CLZ, in agreement with the results of previous studies on rhodopsin [16], [17], [20], cannabinoid CB2 [21] and 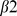-adrenergic receptors [19], [22], where changes in hydration and water-mediated interactions were suggested as relevant contributors to ligand selectivity and receptor activation. A similar role of water molecules in the interaction between the antagonist naltrindole and a 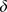-opioid-receptor is suggested in the recent work of Granier *et al.* reporting the first crystal structure of a 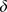-opioid-receptor [27].

The analysis of the local parameters pinpoints larger changes in the presence of DSM than of CLZ. Although smaller than CLZ, DSM is more able to dynamically affect the features of the relevant helices also because of an increased effective volume due to a prolonged interaction with waters. The reduced steric hindrance of DSM with respect to CLZ allows the former to sample a second Basin (Basin 2) and to have a wider interaction interaction pattern than CLZ though not characterized by a particularly pronounced interaction. The outcomes of these dynamical network (including at large the interactions with water molecules) are larger perturbations of the local parameters of the key helices induced by DSM than by CLZ with respect to the apo form (see Table 2). Our results suggest that the different pharmacological activity of the two compounds should be ascribed to change in orientation of the helices, consequence of the different regions sampled by the two compounds.

## Conclusions

Using metadynamics simulations complemented by standard MD runs we studied the interaction of two compounds, clozapine and desmethylclozapine, inside a 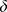-opioid receptor. The different activity of the compounds was traced back to subtle differences in the dynamical interaction pattern with residues and solvent molecules. Despite the two compounds differ only by a methyl group, our simulations highlight many differences in their behavior inside the 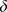-opioid receptor. CLZ, the less active drug, exhibited, according to our simulations, a reduced number of interactions characterized by a long life time with very few residues and the absence of long-time-resident solvent molecules. DSM, the most active compound, benefits from the presence of long-time-resident waters that mediates the induction of structural changes in the structure of the receptor and from a direct interaction pattern with a large number of residues. In support of the experimental data [37], [38], our simulations highlight that DSM induces larger deformations in the 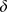-opioid receptor than CLZ. However, the perturbations do not produce particular changes in the secondary structure but essentially affect the relative orientations of the helices. It is worth noting that the more pronounced structural perturbation are induced by the smaller drug, i.e., DSM, probably due to a large network of interacting residues to which the water-mediated interactions should be added. These differences might account in a subtle way for the different activity of the two compounds. In an effort of extracting some general hints from our study we can conclude that in a GPCR as a 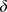-opioid receptor there is a tight coupling among structure, dynamics, interaction with the solvent toward the onset of conformational changes upon activation, which cannot be accounted for by lock-and-key models as well as by induced fit pictures. Additionally, the extension of the conformational changes is still under debate and computational studies at molecular level are in the position of complementing and supporting experiments aiming at clarifying the crucial issue of the ligand-receptor interaction. Moreover, the inventory of the relevant interactions involving the two compounds might serve as a guide for single-point or combined mutations to test the specific role of receptor residues. Such information should open more paths to design efficient compounds since more degrees of freedom are now available. At this stage computational studies at molecular levels will be strongly required in order to suggest design protocols that contain a rational of the several aspects highlighted in the present study.

## Materials and Methods

### Homology model

The structural model of the 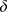-opioid receptor, shown in Figures 1a and b, was built by comparative modeling (i.e. by means of MODELLER 7v7 [67]), by using as a template the crystal structure of dark rhodopsin (PDB code: 1U19 [11]). All the receptor portions but the N-term and C-term were modeled (i.e. 47–333 sequence) because of the low sequence similarity between target and template proteins. Additional problems related to lack of sequence similarity between rhodopsin and 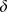-opioid receptor reside in the third extracellular loop (i.e. EL3), which is expected to be longer in the 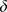-opioid receptor compared to rhodopsin. We attributed such extra-sequence to helix 7 rather than to the unstructured loop, by adding external 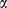-helical restraints to the amino acid stretch 294–302. In this way, the N-term of helix 7 in the target model was two-turn longer compared to the rhodopsin template. Consistent with this result, retrospective analysis shows that the crystal structures of the CXCR4 chemochine receptor of the peptide GPCR subfamily are indeed characterized by a two-turn extension of the extracellular end of helix 7 [68]. The non-structured bridge between helix 6 and the extended helix 7 was finally modeled by a de novo protocol for loop modeling implemented in MODELLER [69]. The employment of this modeling strategy required deletion of almost the entire EL3 in the rhodopsin template (i.e. the 281–286 stretch).

From the same sequence alignment, 50 different models were achieved by randomizing all the Cartesian coordinates of standard residues in the initial model. One model of the 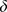-opioid receptor was thus selected, which was characterized by the lowest degree of restraint violations (i.e. the lowest MODELLER Objective Function). Such model was finally subjected to adjustment of side-chain rotamers when in non-allowed conformation.

### System setup

The model of the 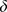-opioid receptor was inserted in a POPC phospholipid bilayer, solvated with 7,000 water molecules, and neutralized by adding 13 chloride counterions [70]. The global size of the system consisted of 35700 atoms. Subsequently, from the X-ray structure of clozapine [71] reported in Figure 1 c we built the atomic configuration of desmethylclozapine replacing the methyl group with a hydrogen atom (see Figure 1 d).

The potential function for our system was built using the parm99 AMBER force field [72], [73] for the receptor, the TIP3P model for water [74], and the Aqvist parameters [70] for the ions. We developed the force-field parameters for POPC, clozapine (CLZ), and desmethylclozapine (DSM) following the Amber protocol. Starting from the initial structure for the three molecules, a geometry optimization was performed using the Hartree-Fock (HF) basis set HF 6–31 G* with the NewChem package [75], [76]. The molecular electrostatic potential was generated at HF 6–31G* level and the related RESP atomic charges were fitted imposing a tollerance of 10^−6^ a.u with respect to the HF results.The GAFF force-field [72], [73] was used to describe bonded and van-der-Waals interactions. Concerning CLZ and DSM, we protonated the atom N4 of both compounds, yielding a charge of +1.

The initial geometry of the system was minimized using the conjugated gradient scheme. The setup, the analyses as well as the atomic-level figures, were performed using VMD [77].

### Molecular dynamics simulation

Once the system was heated to 300 K removing the restraints on the protein, we performed NPT MD simulation to equilibrate the volume of the system and verify the stability of the receptor. Periodic boundary conditions were applied on the x-y plane. Electrostatic interactions were evaluated using the soft particle mesh Ewald schemes [78] with 96×96×96 grid points, 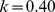
Å^−1^, and a cutoff of 10 Å, as for the Lennard-Jones energy terms. We used a MTS Respa integrator [79] with five shells, with a time step, respectively, of 12-4-2-1-0.5 fs in conjunction with the SHAKE algorithm [80] to keep bond lengths involving hydrogens fixed. The simulations were done at 0.1 MPa and 300 K with Nosè thermostat [81]–[83]. All the simulations were done using the program ORAC. [84], [85] After 20 ns of simulation (first 15 ns applying hisotropic pressure, last 5 ns applying anisotropic pressure of 1 atmosphere) the volume of the system reached a constant value and the resulting box has the following parameters: Z = 85.13 Å, Y = 84.75 Å, Z = 79.89 Å, 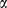 = 90.76°, 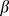 = 90.47°, 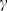 = 60.61°. The RMSD of the receptor stabilized at a value of 3.3 Å. On this equilibrated system we docked manually CLZ in the binding site, for the first system, and DSM for the second system to study the ligands-receptor interaction and ligand exit from the receptor. In these positions both compounds are linked to Asp 128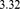, an important residue involved in the recognition of biogenic amines by GPCR [44]. The receptor with the ligands was re-equilibrated following the same procedure described above.

### Metadynamics

The exit process of a ligand in a receptor occur on time scale (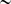
100 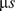) [86], [87] that is not within the reach of standard MD simulations. To overcome this problem we used the metadynamics algorithm [39], [40], [62], [88]–[91]. We briefly review the basic features of the metadynamics method and the protocol we used to study the ligand-receptor interaction processes [92].

The algorithm requires that the process under investigation can be represented using a small sets of reaction coordinates, called also collective variables (CVs), to be defined a priori and be process-dependent. These CVs are complex functions of selected degree of freedom, such as a geometrical coordinate (distances, angles, dihedrals). When the slow evolution of the process can be attributed principally to these few CVs, the artifice is to accelerate the time evolution of the CVs. In our case we chose to sample more efficiently the space defined by the distance between the centers of mass of the compound (either CLZ or DSM) and of the receptor, d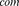, and the orientation of the long axis of the compounds with respect to the receptor axis, 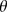.

In other words, the gist of the method is to identify the variables that are of interest but difficult to sample. These variables are functions of the atomic coordinates of the system, and they will be denoted 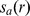. A history-dependent dynamics is constructed in the space of these variables, designed to compensate, as the simulation proceeds, the underlying free energy 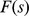. This allows an iterative reconstruction of 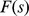 by biasing the dynamics of the collective variables with a history-dependent term [40], [89]–[91]:
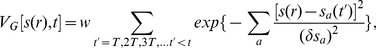
(1)where the time interval 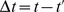 between the placement of two successive Gaussians, the Gaussian width 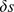, and the Gaussian height 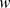 are free parameters that affect the efficiency and the accuracy of the algorithm. The component of the forces coming from the Gaussian will discourage the system from revisiting the same spot, accumulating in the free-energy wells, and allowing the system to migrate from well to well, while all the other coordinates are maintained near equilibrium. In ideal conditions, after a long time the sum of the Gaussian terms will compensate the underlying 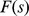 and the system will be free to diffuse on a flattened landscape. In our simulations we choose to add a Gaussian every 4 ps with an height of 2 kj/mol and a width of 5° for 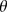 and 0.5 Å for d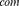.

### Analysis of the interactions with solvent and protein

In each basin of the FESs we performed additional standard MD simulations of 70 ns saving the coordinates every 4 ps to characterize in more details the interaction pattern. In particular, these standard MD simulations were used to evaluate interactions between the water molecules and the two compounds. The survival probability for the water molecules in three different time regimes (fast, medium, slow) bound to the compounds was calculated as mentioned by Sterpone et al. [54]. We then used the following function:
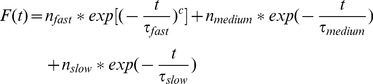
(2)to fit the survival probability and extract the different temporal scales and the associated number of water molecules. In Eq. 2, the first term corresponds to fast regime (around 10 ps), the second to medium regime (around 100 ps) and the last term to slow regime (greater than 1.000 ps). The variables 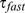, 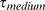, and 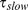 are the different relaxation times and 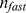, 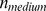, 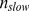 correspond to the average number of water molecules in the three different regimes.

The hydrogen bonds (Hbond) contacts between the substrate and the transporter were defined using cutoff values of 3.0 Å for acceptor-donor distance and 130° for acceptor-donor angle. Hydrophobic contacts (Hphobic) are counted when nonpolar atoms are separated by at most 4.0 Å.

### Binding free energy calculations

**Figure 7:**
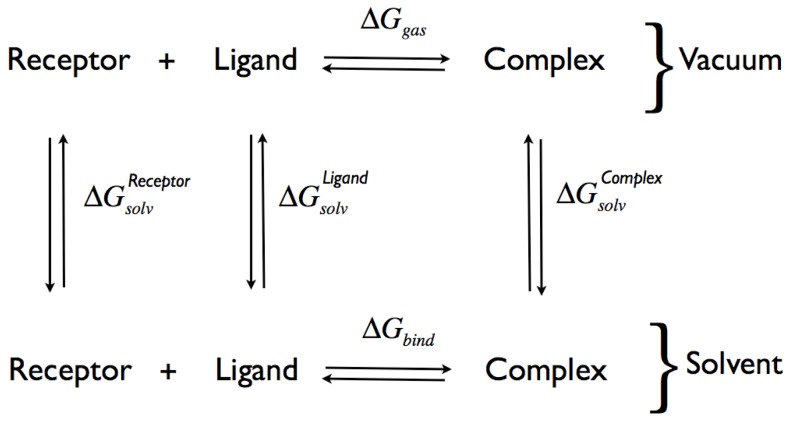
Thermodynamic cycle used for the computation of the free energy of binding. The free energy is decomposed in contributions coming from the solvation energy of the reactant and products, and from the binding free energy *in vacuo*.

Binding free energies for both ligands were estimated by combining the thermodynamic cycle of Figure 7 and MD simulations. Following this approach [93] the binding energy is estimated splitting the calculation in the evaluation of the binding energy *in vacuo* and the effect of solvation on each state of the system: the complex, both the ligand and the protein separately. The equation associated with the thermodynamic cycle is the following:
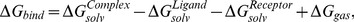
(3)where 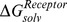, 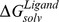, 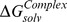 are the solvation free energies of the protein, the ligand, and the complex, respectively, while 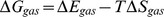 is the binding free energy for the complex *in vacuo*. The two terms contributing to 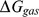, i.e. 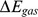 and 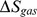, represent the energy of binding and the binding entropy *in vacuo*

The MM/PBSA protocol [94]–[96] implemented in the Amber11 package [97] was used to calculate the solvation energies by solving the Linearized Poisson-Boltzmann equation using dielectric constants of 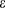 = 1 and 80 to reproduce the *in vacuo* and *in solvent* conditions, respectively [98]. The binding energy 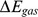 was estimated using the force field used also for the simulation. Finally, we evaluated 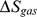 within the framework of the Quasi-Harmonic approximation [47], whose details and limits are deeply discussed in the literature [47]–[53].

For MM/PBSA calculations we extracted one structure every 600 ps from three 70 ns-long MD trajectories [CLZ in Basin 1 (simulation 1) and DSM in Basin 1 (simulation 2) and Basin 2 (simulation 3)] in order to get uncorrelated snapshots for the calculation. We used 116 independent structures to obtain all solvation-related quantities and the binding energy in Eq. 3. The entropy estimations were calculated during the whole 70 ns of the production MD run using all saved frames (35000). As entropy is a time-dependent parameter, 70 ns were chosen to obtain as much convergence as possible [93]. To increase the validity of the Quasi-Harmonic approximation, we evaluated the contribution separately for each minimum of the ligand in the receptor.
